# Functioning and time utilisation by female multi-purpose health workers in South India: a time and motion study

**DOI:** 10.1186/s12960-018-0327-3

**Published:** 2018-11-26

**Authors:** Samiksha Singh, Neha Dwivedi, Amol Dongre, Pradeep Deshmukh, Deepak Dey, Vijay Kumar, Sanjeev Upadhyaya

**Affiliations:** 1 Indian Institute of Public Health-Hyderabad, Public Health Foundation of India, Plot #1, Amar co-op society, Kavuri Hills, Madhapur, Hyderabad, 500033 India; 2 UNICEF Hyderabad Field Office, Hyderabad, India; 3 Department of Community Medicine, Sri Manakula Vinayagar Medical College and Hospital, Pondicherry, India; 4 Department of Community Medicine, All India Institute of Medical Sciences, Nagpur, India; 5 Centre for Economic and Social Studies (CESS), Hyderabad, India

**Keywords:** Multi-purpose health workers, Health workers, Community health services, Personnel management, Time and motion study, India

## Abstract

**Background:**

Auxillary nurse midwives (ANMs) are the most important frontline multi-purpose workers in rural India. This study was conducted to assess the spectrum of service delivery, time utilisation, work planning, and factors affecting functioning of ANMs in South India.

**Methods:**

We conducted a time and motion study in three districts across two states in South India. The districts selected in such a manner that they had a considerable tribal population. We conducted multi-stage sampling to select ANMs. We directly observed 43 ANMs consecutively for six working days and in-depth interviewed all selected ANMs, their supervisors, medical officers, and district health officials. We conducted an FGD to substantiate the findings from observations and interviews. Observation findings were analysed under three broad domains: (i) programme activities, (ii) programme support activities, and (iii) other work. Time spent was calculated in median (interquartile range, IQR) minutes/ANM per week or day. Qualitative data were coded and analysed using grounded theory, and appropriate themes and sub-themes were identified.

**Results:**

ANMs worked for median 7 h a day (7:10 h, non-tribal; 6:20 h, tribal). There is variation in the hours of work, the pattern of service provided and time utilisation across days of a week. ANMs spent 60% of their on-job time on programmatic activities (median 22:38 h; IQR, 20:48–27:01 h) in a week. Emphasis is more on home visits, universal immunisation, antenatal care, school health, and seasonal diseases. ANMs spent negligible time on non-communicable diseases, adolescent health, nutrition, etc. ANMs spent the remaining time in program support activities, such as meetings with seniors, community meetings, and other non-health related work. There are no renewed job description, work plans, and supervision guidelines, even with newly added programs and tasks. ANMs prioritised work as per the priorities set by the supervisors and leaders. Health administration often disrupts the regular functioning of ANMs for training, meetings and other ad hoc work.

**Conclusion:**

ANMs are overworked; they often multi-task and fail to deliver efficiently. The administration needs to re-assess the workload. The administration may reduce expected work, provide strong supervisory support, and make conscious efforts to pose fewer disruptions in regular working of ANMs.

## Introduction

Qualified and motivated human resources are essential for adequate health service provision [1]. A global strategy on human resources for health realised that low- and middle-income countries face severe challenges in ensuring a sufficient, fit for purpose and fit to practice health workforce [2]. An insufficient, inefficient and ineffective health workforce compromises the health status of communities. Simultaneously, burdening the health workforce with multiple responsibilities especially in resource-poor systems also decreases efficiency [3].

A study on human resources in India (2011–2012) found that there were on average 3.4 doctors and 3.3 nurses or midwives per 10 000 population. There was large disparity between urban (doctors, 9.1 per 10 000 and nurses, 7.2 per 10 000) and rural areas (doctors, 0.8 per 10 000 and nurses, 1.3 per 10 000). Rural population relied more on a nurse or midwife for primary health care. However, of the existing nurses or midwives, more than half had not achieved minimum qualification and the scenario was worse in rural India [4]. In India, there is no separate midwifery cadre but the general nurses and auxillary nurse midwives (ANMs) are considered a mix of nursing and midwifery.

ANM, a cadre established by the British in India to provide midwifery care, is the most important and most peripheral public health functionary in rural India. These frontline health workers are now female multi-purpose health workers who provide primary promotive and curative health care for almost all the national health programs through a sub-centre (SC, the most peripheral health centre in rural India) and outreach activities in catchment villages. In India, the National Health Mission (NHM) brought in the provision of two ANMs for every SC to reduce the burden on one ANM and to strategically distribute the work between them for improving availability and coverage of health services. The introduction of accredited social health activists (ASHAs) by NHM for every village aimed towards facilitating the community outreach and enhancing beneficiary coverage and support to ANMs [5]. Male multi-purpose health worker (male-MPHW) although available only at some SCs are closely involved in various disease control programmes [5, 6].

There are only a few studies that have assessed the work load of ANMs and their time utilisation in India, especially after the provision of the second ANMs and ASHAs, and the depleting male-MPHW workforce. We aimed to analyse the time spent by ANMs on specific activities as per their job description, work planning and the factors affecting the service delivery.

## Methods

We conducted a large study that assessed the functioning and time utilisation by frontline health workers, namely ANMs, male-MPHW, and ASHAs, and studied inter-cadre task sharing and coordination. This manuscript mentions methods and results with the focus on ANM cadre. Details of the full study are mentioned in the protocol paper published elsewhere [7]. Ethics approval was obtained from the institute’s ethics committee (TRC/IEC-127/2015) and authorities.

### Study design and study area

This was a cross-sectional study using mixed-methods (observations using ‘time and motion’ approach, interviews and focus group discussion (FGD)). The study was conducted across three randomly selected districts from two states in the south of India—Andhra Pradesh (Srikakulam and Chittoor) and Telangana (Khammam). The districts are predominantly rural but also have a substantial tribal population—6.2%, 3.8% and 27.4%, respectively in Srikakulam, Chittoor and Khammam [8]. Tribal terrains in Srikakulam were predominantly hilly while in Khammam it was plains. Table 1 presents a few maternal and health indicators of the selected districts from the National Family Health Survey-4 (NFHS-4) 2015–2016 [9–12].

**Table 1:** Maternal and child health indicators of three study districts (in %): National Family Health Survey-4 (NFHS-4) 2015–2016 [9–12]

Indicator	Srikakulam	Chittoor	Khammam	India
Family planning				
Current use of any family planning method	67.7	61.3	69.1	53.5
Unmet need of family planning	6.7	3.3	4.5	12.9
Fertility				
Births to women 15–19 years out of total births	14.0	14.0	16.9	7.9
Antenatal care				
Pregnant women who had antenatal check-up in first trimester	82.2	72.0	83.9	58.6
Pregnant women who had at least four antenatal check-up visits	72.7	70.4	79.2	51.2
Delivery care				
Institutional	91.2	94.0	94.2	78.9
Government	42.3	53.0	32.4	52.1
Private	48.9	39.0	61.8	26.8
Child received full vaccination	59.2	67.6	62.4	62.0

### Study population

The study population constituted of two cadres of ANMs working at SCs. ANM-1 are senior and permanent staff while ANM-2 are newly recruited contractual staff (post year 2005). There is also a provision of ANM-3 under funding from the European Commission if the geographical coverage area of SC is large.

### Sample size and sampling

We conducted multi-stage stratified sampling. At the planning stage, Andhra Pradesh and Telangana were a combined state that partitioned in June 2014. We selected three districts, one from three regions of the combined state of Andhra Pradesh. We stratified the districts into non-tribal and tribal clusters, and randomly selected one cluster of each type. Chittoor did not have a substantial tribal population, thus we randomly selected two non-tribal clusters. From each of the sampled clusters, we selected one primary health care centre (PHC) randomly, and within each sampled PHC, we selected four SCs. We listed all SCs with distance from PHC and sampled one farthest, one closest and two intermediate distant SCs—ANMs were required to frequently visit PHC and distance from PHC would affect time utilisation. We recruited all the ANMs posted at the selected SCs. In total, across three districts, we sampled 24 SCs which had 43 ANMs (21 ANM-1s and 22 ANM-2s).

### Data collection and study tools

We obtained permissions from the administration of the respective locations before initiation of the study.

#### Quantitative

We directly observed 43 ANMs from home to home consecutively for 6 days (Monday–Saturday) recorded activities and time spent, using a structured checklist. We developed a software app to capture information and used global positioning system-enabled tablet computers to more accurately document time, multi-tasking and movement of ANMs in the field. We silently observed the participants by shadowing them home to home and without disturbing the regular functioning of the ANMs. Whenever the client was not comfortable with the presence of the observer, the observer stayed out and recorded the findings with the information provided by the ANM. Such instances were rare and only during home visits.

We developed an observation checklist using an inductive approach based on exploratory field visits done during the formative stage of the study. Visits yielded exhaustive data with a detailed account of uncategorised activities performed by ANMs. We analysed this data and simultaneously explored the job responsibilities assigned by the authorities and extracted important information. Using this information, we identified appropriate themes and codes and developed an exhaustive list of categories and sub-categories of work with specific activities within each sub-category. We piloted the checklist twice and finalised it before translating it into a software application. The final observation checklist consisted of major categories as listed in Table 3. These categories were further sub-divided into sub-categories with an activity list against it. For example, under the category ‘direct service to beneficiary’, 17 sub-categories were devised like adolescent health, blindness/cataract, camp work and child health.

#### Qualitative

Similar to checklists, we developed qualitative interview schedules by a re-iterative process and identified key areas of inquiry. Semi-structured interview guides for ANMs, supervisors, medical officers, and district officials were originally framed in English and then translated into local language and were culturally adapted during pilot tests. Interviews with ANMs gathered information on socio-demographic and economic profile, physiologic status, functioning, work planning, supervision, training requirements, etc. Later in the study, we conducted one FGD with nine ANMs to substantiate the findings from observations and interviews. Interviews with supervisors and officials gathered information on their understanding and perceptions of ANMs’ work and suggestions to improve the efficiency of work.

We obtained written consent from the participants before conducting any observations and interviews. All the selected participants consented for the study. Trained observers conducted observations and interviews, covering one PHC at a time and moving to the next PHC subsequently. Observations and interviews were done in a participants’ natural work setting (field or health facility). Field leads directly supervised the observers and also maintained notes for other observations. The trained field leads also interviewed the health supervisors and officials and conducted an FGD. A dedicated central coordinating team monitored the overall data collection, data management and analysis. We did not record any personal identifiers and stored data in a password-protected system, which was accessible only to the study leads and the manager.

### Data analysis

Quantitative data was managed and analysed using Microsoft Excel 2010. Observation findings were presented under three broad domains: (i) programme activities, comprising of service delivery, recording and travel within the field, (ii) programme support activities, comprising of training, meetings, administrative work and activities outside job description, and (iii) other work, comprising of waiting, personal work and uncategorised. Time spent was calculated in median (interquartile range, IQR) minutes/ANM per week or day. We also did a stratified analysis for tribal and non-tribal, and ANM-1 and ANM-2; however, only relevant information is presented in the text. Interviews yielded both quantitative and qualitative information.

Qualitative interviews and FGDs were transcribed and translated into English, where required. Qualitative data from interviews and information from FGDs were coded and free listed using Anthropac 3.2.2 software. Data were coded and analysed using grounded theory and appropriate themes and sub-themes were identified. Qualitative information from all the stakeholders was compiled and presented to understand the overall picture.

## Results

We observed 43 ANMs (13 from Srikakulam, 15 each from Chittoor and Khammam) for a span of six consecutive days (Monday to Saturday). During observations in one of the SCs, a Friday was a public holiday thus eight ANMs did not work that day. In all, we observed 249 ANM days.

### Social profile of ANMs

Twenty-seven ANMs were between 20 and 35 years, and 16 above 35 years. All ANMs were educated up to the minimum eligibility criteria of high school followed by the ANM diploma course. ANM-1s completed their course about 15 years back and ANM-2s had completed their course about 6 years back. Two ANMs had also done a higher nursing course and were awaiting promotions. A total of 36 ANMs were married, four were widowed and three were unmarried. Of the 43 ANMs, only five were staying in SC quarters. ANMs were not residing in SCs or nearby due to non-availability of quarters, or non-conducive living conditions, not a safe location, or choice of family.

### Time spent home to home

**Fig. 1:**
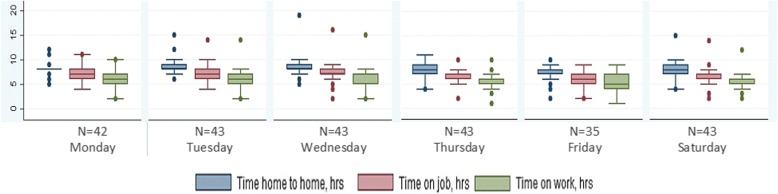
Median time spent by ANM from home to home, on job and on designated work in hours per ANM by day of the week

Table 2 and Fig. 1 show the median (IQR) time spent between home to home, on job including within field travel, and time spent on actual work. ANMs spent median 8:04 h (non-tribal 8:09 h; tribal 7:58 h) from home to home. Time was spent more on Wednesdays and Thursdays and least on Fridays and Saturdays. On any given day, ANMs travelled more than 2 h in total. Thus effectively an ANM was in client contact or meetings for only median 5:52 h (non-tribal 6:06 h; tribal 5:01 h). ANMs from tribal areas left from duty early due to safety concerns and rainy weather (at the time of data collection, monsoons had set in tribal Srikakulam district). ANM-1s and ANM-2s spent almost equivalent median time on actual work (5:54 h and 5:49 h respectively) and on within field travel (0:57 h and 1:07 h respectively). Tribal areas comprised of either hilly remote terrain or scattered tribal hamlets. ANMs walked to such areas due to unavailability of public transport. They were sometimes supported by male-MPHWs, villagers or family members to visit remote areas.

**Table 2:** Time in median hours (IQR) per ANM per day from home to home (*N =* 43; hh:mm)

Particulars	Total	Non-tribal PHC	Tribal PHC	1st ANM	2nd ANM
Travel from home to home	8:04 (7:18–8:49)	8:09 (7:30–8:48)	7:58 (6:53–9:00)	8:04 (7:02–8:37)	8:01 (7:25–9:00)
Travel from home to work place and work place to home	1:04 (0:36–1:36)	1:00 (0:32–1:26)	1.37 (0:58–2:06)	1:12 (0:37–1:41)	1:02 (0:37–1:29)
Time on job (including within field travel)	7:00 (6:08–7:31)	7:10 (6:27–7:42)	6:20 (5:16–7:09)	6:54 (6:00–7:20)	7:09 (6:12–7:57)
Within field travel	1:02 (0:36–1:36)	1:02 (0:38–1:37)	1:02 (0:25–1:42)	0:57 (0:30–1:25)	1:07 (0:39–1:52)
Work time	5:52 (4:45–6:44)	6:06 (5:11–6:48)	5:01 (3:49–6:25)	5:54 (4:54–6:40)	5:49 (4:35–6:49)

### Time spent on activities

In a week, ANMs spent about 60% of their on-job time on programme activities, 22% on programme support activities and 18% on other activities. ANMs devoted time to all the three categories during all days of the week. We observed that ANMs did not have well-defined lunch breaks. Lunch was often clubbed with field- or centre-level activities, or personal work. Table 3 presents the time spent in broad categories and sub-categories. In tribal PHCs, the ANMs spent much less time in programmatic activities including direct service delivery compared to ANMs in non-tribal PHCs. ANM-1s and ANM-2s had clearly demarcated geographical areas and each one of them performed a similar set of activities in their areas of responsibility.

**Table 3:** Time spent within broad categories and sub-categories of work, *N* = 43 (in minutes)

Particulars	Total minutes/week				
	Count *N* = 43 (%)	Range		Median (IQR)	Avg./week/ANM
		Min	Max		
1. Programme	43 (100.0)	742	2 368	1 358 (1248–1 621)	1 441
1.1 Direct service to beneficiary	43 (100.0)	234	1 405	586 (439–872)	664
Adolescent health	4 (9.3)	0	8	0 (0–0)	4
Blindness/cataract	2 (4.7)	0	32	0 (0–0)	21
Camp work	11 (25.6)	0	221	0 (0–14)	99
Child health	15 (34.9)	0	13	0 (0–6)	8
Communicable diseases	18 (41.9)	0	162	0 (0–4)	20
Curative care	7 (16.3)	0	29	0 (0–0)	17
Family planning	6 (14.0)	0	43	0 (0–0)	17
IEC activities amongst group(s)	13 (30.2)	0	35	0 (0–8)	15
Maternal health	24 (55.8)	0	197	8 (0–22)	43
National health day	3 (7.0)	0	71	0 (0–0)	35
Non-communicable diseases	12 (27.9)	0	73	0 (0–3)	17
Nutrition	2 (4.7)	0	4	0 (0–0)	4
School health	27 (62.8)	0	259	25 (0–56)	70
Seasonal diseases/epidemic outbreaks	34 (79.1)	0	121	10 (3–38	34
Universal immunisation day	30 (69.8)	0	777	257 (0–470)	387
Home visits	42 (97.7)	0	727	200 (105–360)	250
Others	31 (72.1)	0	534	51 (0–89)	95
1.2 Records and reports	43 (100.0)	12	886	422 (236–537)	386
Beneficiary records	12 (27.9)	0	39	0 (0–4)	16
Computer data entry	15 (34.9)	0	268	0 (0–43)	104
Health pro formats	1 (2.3)	0	6	0 (0–0)	6
Registers	41 (95.3)	0	581	232 (101–413)	264
Reports	33 (76.7)	0	623	21 (5–100)	102
Others	21 (48.8)	0	135	0 (0–18)	31
1.3 Travel to and within field	43 (100.0)	101	893	352 (289–450)	391
2. Programme support	43 (100.0)	97	981	518 (341–670)	523
Trainings	07 (16.3)	0	331	0	307
Meetings/discussions with co-workers or village community	43 (100.0)	53	428	214 (150–316)	225
Meetings/discussions with seniors	43 (100.0)	4	547	146 (90–211)	157
Non-health but work-related activities	17 (39.5)	0	121	0 (0–14)	39
Administrative work	43 (100.0)	17	270	66 (39–91)	75
3. Other work	43 (100.0)	220	787	426 (335–484)	420
Waiting	36 (83.7)	0	210	59 (11–114)	87
Miscellaneous: personal work	43 (100.0)	79	520	307 (249–365)	309
Others/uncategorised	22 (51.2)	0	333	2 (0–52)	76
Total on-job	43 (100.0)	1 083	3 948	2 439 (2129–2 598)	2 384

Count gives number of ANMs performing the respective activity. Time for ANMs not doing activity was considered as 0 while computing range and median (IQR). Average time was estimated per week for only those who performed the activity

#### Programme activities

ANMs spent median 1358 min in a week (IQR, 1248–1621 min) on programme activities. Within programme activities, most time was spent on direct service delivery (median 586 min; IQR, 439–872) which was more on Wednesday, Thursday and Saturday. In these 3 days, ANMs practiced core service delivery wherein universal immunisation day (UID) was organised at SCs on Wednesdays; school health and field outreach on Thursdays and ‘nutrition and health day’ and field outreach on Saturdays. They maintained records and reports mostly on Mondays.

The top five activities performed were home visits, seasonal diseases/epidemics/outbreaks control work, universal immunisation, school health and maternal health, performed by 42, 34, 30, 27 and 24 ANMs respectively. In terms of time spent, apart from UID (median, 257 min in a week) and home visits (median, 200 min in a week), time spent on other vital components of direct services was much lower, almost negligible in few cases (Table 3). During home visits, ANMs provided services for maternal and child health, general health, counselling, medicine distribution, etc. For seasonal diseases/epidemic outbreaks, ANMs provided services such as identification of cases, referral and counselling for fever, malaria, dysentery, etc. We observed that ANMs did a lot of multi-tasking during UIDs and home visits. On UID, ANMs simultaneously did other activities like general health check-ups, medicine distribution, antenatal check-ups, counselling, generating awareness, maintaining records and personal phone calls. While travelling to and within the field, ANMs enquired for new cases, conducted community mobilisation, counselling and meetings with ASHAs apart from attending to specific cases in home visits.

Other than home visits, only 24 ANMs were observed to provide maternal and child health services through centre-based activities. The time spent by these 24 ANMs for maternal health ranged from 1 to 197 min per week. Under maternal health activities, ANMs conducted antenatal women’s registration, antenatal women’s routine check-up (blood pressure, height, weight, haemoglobin testing), birth and death registration, postnatal women’s routine check-up, counselling, etc. However, it was observed that ANMs found difficulties in performing antenatal check-up mainly because of inadequate training. Few ANMs struggled with basic functions like haemoglobin testing and accurately recording the readings. None of the ANMs conducted deliveries at any of the SCs. ANMs referred pregnant women for delivery to a PHC or directly to the higher centre. Child health activities (apart from UID) involved identification, referral and accompanying the sick child, follow-up with drop outs of immunisation, curative care and counselling.

Only three ANMs participated in nutrition and health day organised with support from ASHAs and Anganwadi workers. This is supposed to be at least a half a day activity; however, these three ANMs spent only an average of 35 min on the activity. Eleven ANMs participated in camps such as medical health camps and family planning operation camps. Time spent on Information Education and Communication (IEC) activities was much lower within which time was mostly spent on IEC for child health, mobilisation of beneficiaries for any camp(s) and others like communicable diseases and maternal health issues. Twelve ANMs provided services for non-communicable diseases, through mostly a government run mobile van service, on pre-decided fixed days.

There was slight variation in functioning of ANMs across districts. ANMs from Chittoor spent some time on nutrition and family planning which other district ANMs did not. ANMs from Chittoor spent more time in centre-based care; ANMs from Khammam tribal PHC spent more time in service delivery through home visits, while ANMs from Srikakulam seem to have a balance of centre-based service delivery and home visits. We also observed two big activities being conducted apart from routine work: first, a mass immunisation campaign named ‘Mission *Indhradhanush*’ in Khammam which required staff to search under-five children house to house and complete their immunisation and, second, training of ANMs for use of tablets for recording and reporting information under a ‘mother and child tracking system’ in Chittoor. During these activities, the regular work was not undertaken.

In total, ANMs spent a median 422 min (IQR, 236–537 min) per ANM per week on records and reports. ANMs maintained at least 18 kinds of different registers. ANMs in Khammam maintained extra books to record data and noted information in an organised way convenient to them. ANMs expressed that this helped them in quick reporting to the management and keep track of field-level requirements. Apart from registers, there were various kinds of reports (weekly, monthly, quarterly, etc.).

##### Travel within the field

We also estimated time spent on travel to reach the field sites and return from there (excluding time spent in service delivery). This was a median 39 min on Tuesday and about 60 min on rest of the days. ANMs stated that they had limited time for field related work. Remote locations from PHC/SC were often either left out or were covered less frequently.

#### Programme support activities

In a week, ANMs spent median 518 min (IQR, 341–670 min) on programme support activities. Seven ANMs (from Srikakulam) attended training for the ‘mother and child tracking system’ and spent an average 307 min per week for this. All 43 ANMs utilised their time for meetings/discussions with co-workers or the village community which was often done during their time of travel to the field. The time spent on meetings/discussions with seniors was only between 7 and 15 min a week. Seventeen ANMs were engaged with health-related activities other than their prescribed job responsibilities. ANMs, especially the second ANMs, were mainly engaged in field-level survey activities or outpatient duties at the PHC or at the medicine counter because of the unavailability of the staff nurses or pharmacists.

We observed that ANMs from one of the non-tribal PHCs from Chittoor spent more time in the programme than non-programme functions. This PHC was managed by a trust under public-private partnership and had a dedicated focus on service delivery.

#### Other work

ANMs spent considerable time waiting for patients on designated clinic days. Other time was spent on personal talk on the phone with family/friends, lunch break, sitting and chatting with people, which was beyond the purview of the ANM’s regular work. ANMs spent median 426 min in a week/ANM (IQR 335–484 min) on other work.

### Qualitative results

#### Work plans

All ANMs stated that they planned their work using advanced tour plans. We observed that tour plans did not have a uniform format, and none had any activity-wise listing for service delivery. Only a few plans mentioned fixed day activities such as UID and antenatal check-up. One of the ANMs stated that she used plans devised for the malaria control programme which mandated that each village is visited at least twice a month. None of the tour plans had a buffer for any unforeseen health or non-health activities. ANMs stated that they rarely could follow their advanced tour plan completely. ANMs planning and execution of work plan/tour plan was facilitated by supportive supervision, support from co-workers and support from the community including village groups, leaders, self-help groups and village elders.

#### Job responsibilities

ANMs could not describe their job responsibilities properly. They felt the new programmes and initiatives were a great strain and none of the ANMs understood how to incorporate the guidelines into their overall day to day grassroots-level planning. One ANM stated that she did as much she could and another stated that she had no choice but to deliver whatever is expected by the seniors. It was apparent that there was no holistic plan and ANM’s work was defined by some fixed expectations (e.g. UID and antenatal care), many sporadic activities based on the preferences and needs of the several programmes, and those decided by the district or state administration. Officials felt the tasks assigned to ANMs were doable and they needed to be planned better. ANMs job is to manage routine and ad hoc work by themselves. They felt that supervision and monitoring were largely lacking thus the efficiency of ANMs was poor.

#### Facilitating factors or barriers to utilisation of time

The concept of time management was very abstract for ANMs and only meant a different set of activities and corresponding time spent. The ANMs expressed that the following consumed most of their time: immunisation, home visits comprising, identification of cases and treatment, seasonal diseases/epidemics/outbreak-related surveys, recording and reporting and antenatal clinics. The facilitators and barriers to efficient functioning are described in Table 4.

**Table 4:** Facilitating factors and barriers to efficient working by ANMs

Domain	Facilitating factors*	Barriers*
Interpersonal factors and community related	*Co-worker’s support*	*Lack of community support*
	*Community support*	Lack of co-workers’ support
	*Family support*	Local community beliefs
	School support	Fear of violence and alcoholism in remote tribal areas
	Community awareness	
	Beneficiaries support	Movements of village people for seasonal jobs disrupts continuity of care
	Self-motivation	
	Knowing local language	
Health system-related factors	*Clear work plan*	*Difficult transportation*
	*Ease in transportation*	*Training needs*
	Proper infrastructure, stocks and supplies	*Poor infrastructure, inadequate stocks and supplies*
	Supervision and support from district health system	*Accompanying beneficiaries to higher facilities*
	Work atmosphere	*Vacant positions*
		Low salaries of contractual ANMs
		Carrying heavy vaccine kits
		Multiple health programmes
		Records maintenance
		Online MCTS
		Work pressure
		Sudden meetings
		No ambulance service
		Health facilities in interior locations
Others	Political support	*Extreme climatic conditions*
		Political interference
		Physical ill health
		Emergencies/ outbreaks
		Concerns of family

*Those in italics were most frequently mentioned

ANMs stated enabling factors such as community support, co-workers support, family support and ease in transportation (staying closer to a health facility, availability of public transport, possessing own vehicle, husband dropping her, etc.). ANMs stated that trainings were an important factor for accomplishing their tasks effectively and accurately. ANMs expressed the need for periodic refresher trainings with hands-on exposure and on-job trainings with the support of supervisors. ANMs strongly expressed the need to be trained in some key technical areas such as testing for communicable diseases; testing for haemoglobin; information about new health programmes and initiatives; online mother and child tracking system; records maintenance; maternal and child health-related aspects; new immunisation; adolescent health; testing for non-communicable diseases, emergency medicine and basic delivery and emergency obstetric care.

We probed into coordination with and support from co-workers. The findings were indicative of direct work support between the ANM-1s and ANM-2s. ANM-1 and ANM-2 demarcated their geographic areas and were responsible for all the field-related tasks for that area. ANMs maintained their records for respective populations and compiled the records for the SC. This reduced burden of coverage and documentation on one ANM to half. ANMs also mentioned that on special days such as UID, they distributed the work amongst themselves and received support from ASHAs. ANMs acknowledged that the presence of ASHAs was vital for their functioning in various significant ways like connecting with the community and mobilising them; gathering information about new cases, births and deaths in the village; liaising with village leaders and *Panchayat Raj* Institution (most peripheral rural administrative body) representatives; and support during home visits, special drives and special days like surveys, National Health Day and UID.

ANMs felt that the support of male-MPHW and coordination with him was crucial for their work, but most of the male-MPHW positions were vacant. In Srikakulam’s tribal PHCs where male-MPHWs were present, ANMs described that male-MPHWs helped in managing communicable and non-communicable disease control-related activities while ANMs could focus more on maternal and child health components. With support from male-MPHWs, ANMs could mobilise the community better especially men and the elderly. Male-MPHWs accompanied ANMs to remote areas and often provided transport support. ANMs stated that there were some tribal areas where they did not feel safe and avoided to go if a male-MPHW was not coming along. Anganwadi workers (frontline worker in department of women and child development) were another important cadre that provided support to ANMs for community mobilisation; identification of antenatal women, children to be immunised, postnatal women, eligible couples, seasonal diseases, HIV, Leprosy, etc.; and support during special event days, village-level surveys and health education.

Amongst the barriers, transportation (unavailability of public transport, difficult to reach remote and hilly terrain with no or *kachha* roads, etc.) emerged as the most commonly stated challenge. Other challenges stated were lack of support from community, family and co-workers; ill health of ANM; extreme climatic conditions; meetings (sudden, long duration and timings coinciding with routine tasks); other tasks (surveys, outpatient duties or pharmacy work at PHC, etc.); poor infrastructure; extra work due to vacant positions of co-workers; carrying heavy vaccine kits and records maintenance.

A crucial aspect emerged about the physical health of ANMs. Most ANM-1s were above 40 years of age and they stated that strenuous work, especially during field days, got affected due to ill health. We observed an ANM-2 who was pregnant and continued her field visits efficiently; however, she complained that she was on a contractual post, thus she did not have a liberty to avail of maternity leave. She would have to leave her job for delivery and she feared that she might not get her job back. Salaries of contractual ANMs was about half of the regular ANMs for the same amount of work.

## Discussion

Our study observed that ANMs spent a median of 7 h on the job per day, of which only 60% of the time was spent on direct program activities. With respect to time utilisation by ANMs, we compared our findings with the suggested time in the NHM guidelines (Table 5) [13]. We observed that ANMs spent a median 45 h per week against suggested 42 h per week. There is less scope of increasing ANMs’ working hours but the ANMs who were spending less time should be closely supervised and supported to at least provide 42 h on the job per week. Adequate community health worker management comprising of effective and supportive supervision is known to have had demonstrated tangible impact onto their performance [14–16].

**Table 5:** Comparison of time spent by ANMs in the study with suggested time in guidelines

Core categories of work	Time observed, hours/week	Time suggested, hours/week*
a) Outpatient services at the sub-centre	2	30
b) Services delivered in the outreach mode: immunisation/nutrition and health day/school health/camps, etc	10	
c) Services delivered during home visits and visits to the community	4	
d) In-patient midwifery services of the sub-centre	0	
e) Maintaining records and reports, planning her work, building her capacity, meetings, inter-habitation movement	22	10 to 14
f) Referral of high-risk pregnancies, sick neonates and other emergencies	0	
Others (no such category suggested by the government)	7	–

*Guidelines for ANM work under NHM [13]

ANMs spent only median 16 h per week on service delivery compared to 30 h suggested by NHM (Table 5). However, ANMs stated that two ANMs per SC and support from ASHAs have decreased a lot of their work and increased the coverage in terms of a number of clients they serve. The NHM guidelines for the ANMs suggest that ANM-1 and ANM-2 shall stay at the SC and visit field on alternative days such that one ANM is always present at SC for the first half of every working day. This kind of task division/sharing can be an optimal means of utilising human resources that can reduce the quantum of tasks into one person and enable them to better accomplish service delivery [17–19]. We observed that despite two ANMs at SC and geographic distribution of work, the distances and population to cover are large with respect to the time required to provide promotive and basic curative care through outreach activity. This is even more difficult in sparse habitations, an absence of transportation, low skills and multiple tasks in hand.

We observed that most of the service delivery was only for a few components of two health programs (maternal and child health and communicable disease outbreaks). They provide services for other health programs during home visits and camps or special days. ANMs spent negligible time on programs for non-communicable diseases, specific communicable diseases, adolescent health, nutrition, etc. Apart from direct service delivery, ANMs were required to do several programmatic (records and reports), programmatic support (meetings, trainings, etc) and non-health related activities (election duties, surveys, etc). The latter two are usually not considered while recommending/monitoring ANMs work schedule/plan. ANMs do a lot of multi-tasking to be able to carry these tasks. Studies suggest that multi-tasking by health workers coupled with other systemic factors like lack of staff, skills and supervision compromises on their capacities [20–22].

ANMs were envisaged for midwifery care in rural India, but they were later realised as multi-purpose workers. In the process, their curriculum and trainings also were modified to fit a multi-purpose worker profile. None of the ANMs in our study practiced midwifery at home or at SCs, a few however asked for trainings for basic emergency obstetric care especially in tribal areas. ANMs expressed the pressure of performing for health programmes other than maternal and child health. They required repeat trainings and supervision for several health programs. The quality of trainings provided, practical integration of programs at the grassroots level and hand holding support were key areas to improve the efficiency of the work.

Guidelines in India do not have any standards or scientific estimates of how much time is required by ANMs to perform all the desired tasks of all the health programs to be delivered. As per these guidelines, ANMs should be able to accommodate tasks of all the health programs in their work plans. But the ANMs in our study stated that they were unable to accommodate these in their regular schedule. With the limited number of work hours, any new activity replaces the previous one. ANMs thus perform only those tasks which are the priority or are closely monitored in real time or are demanded on a time to time basis. District administration may call for any training or meeting or ancillary work (reports, surveys, election duties, etc.) without considering its impact on ANMs’ regular work schedule. Although these tasks are in a broad domain of work, frequent untimely disruptions in routine work reduce performance. In low- and middle-income countries amidst a human resources crunch and dual burden of diseases, task shifting and prioritisation with a rational distribution of tasks can be instrumental [20, 23, 24]. In India, this would require adding more numbers to existing frontline health cadres, adding more male-MPHWs, and providing close supervision. Mostly, the administration will need to respect the schedules of the frontline health workers and align their agendas accordingly.

### Strength and limitation

Our study is unique in that it observed the functioning of ANMs and assessed views about it from supervisors at different levels. Although we could only observe 43 ANMs, it was the biggest sample of such studies from India. We used a mixed-methods approach and imbedded inductive philosophy into a deductive approach of data collection. We could have gathered more information and variation if we had observed each ANM for two subsequent weeks. Nonetheless, we observed 249 ANM days that provided us with enough sample data to arrive at important results and conclusions.

## Conclusion

ANMs work for a median 7 h a day and 6 days a week. There is variation in the hours of work, the pattern of service provided and time utilisation. The emphasis is more on antenatal care, universal immunisation, school health and seasonal diseases. ANMs work on an ad hoc basis as per the priorities set by the supervisors and leaders. ANMs are overworked; they often multi-task and fail to deliver efficiently for all the variety of health programs. There are no renewed job descriptions, work plans and supervision guidelines, especially in the scenario of newly added programs and tasks. The health administration expects much work output but disrupts the regular functioning in the name of trainings and other ad hoc work. The administration needs to undertake re-thinking and needs to down-size the work load, add more manpower with strong supervisory support and make conscious efforts to pose fewer disruptions in regular working of ANMs.

## Abbreviations

ANM: Auxillary nurse midwife
ASHA: Accredited social health activist
FGD: Focus group discussion
IEC: Information Education and Communication
IQR: Interquartile range
MCTS: Mother and child tracking system
MPHW: Multi-purpose health worker
NHM: National Health Mission
PHC: Primary health care centre
SC: Sub-centre
UID: Universal immunisation day
